# Non-invasive detection of microvascular changes in a paediatric and adolescent population with type 1 diabetes: a pilot cross-sectional study

**DOI:** 10.1186/1472-6823-13-41

**Published:** 2013-10-05

**Authors:** Sarah P M Hosking, Rani Bhatia, Patricia A Crock, Ian Wright, Marline L Squance, Glenn Reeves

**Affiliations:** 1 John Hunter Children’s Hospital, New Lambton, NSW 2305, Australia; 2 Monash University, Melbourne, VIC 3168, Australia; 3 Discipline of Paediatrics and Child Health, University of Newcastle, University Drive, Callaghan, NSW 2308, Australia; 4 Department Paediatric Endocrinology and Diabetes, John Hunter Children’s Hospital, New Lambton, NSW 2305, Australia; 5 Hunter Medical Research Institute, New Lambton, NSW 2305, Australia; 6 Autoimmune Resource and Research Centre, New Lambton, NSW 2305, Australia; 7 John Hunter Hospital, New Lambton, NSW 2305, Australia; 8 School of Biomedical Sciences and Pharmacy, Faculty of Health, University of Newcastle, University Drive, Callaghan, NSW 2308, Australia; 9 Graduate School of Medicine, University of Wollongong, Wollongong, NSW 2522, Australia

**Keywords:** Type 1 diabetes, Nailfold capillaroscopy, Laser Doppler flowmetry, Retinal vessel analysis, 24-hr ambulatory blood pressure monitoring, Microvascular disease

## Abstract

**Background:**

The detection of microvascular damage in type 1 diabetes is difficult and traditional investigations do not detect changes until they are well established. The purpose of this study was to investigate the combined ability of nailfold capillaroscopy, laser Doppler flowmetry, retinal vessel analysis and 24-hr ambulatory blood pressure monitoring to detect early microvascular changes in a paediatric and adolescent population with type 1 diabetes.

**Methods:**

Patients aged between 8 – 18 years with type I diabetes and no other autoimmune conditions were studied. The participants underwent the above cardiac and vascular investigations in a single three-hour session. Standard parameters including HbA1c were also investigated. Associations between all parameters were described by correlation analysis. Fisher’s exact and t-tests determined the association with clinical findings.

**Results:**

26 participants were recruited. The mean HbA1c was 8.1% (SD ± 1.1) with a mean duration of type 1 diabetes of 7.9 years (SD ± 3.4). Three participants had microalbuminuria and one had early signs of retinopathy. Participants with microvascular complications had more avascular areas on nailfold capillaroscopy (p = 0.03). Recent HbA1c was positively associated with the number of nailfold microhaemorrhages (p = 0.03) Decreased baseline perfusion by laser Doppler flowmetry was associated with increased capillary density (p = 0.001) and an increased number of microaneurysms (p = 0.04) on nailfold capillaroscopy.

**Conclusions:**

This pilot study has shown that in children and adolescents with established type 1 diabetes, abnormal microvasculature can be detected by these investigations. These markers were also positively associated with evidence of suboptimal diabetes control as assessed by HbA1c. Further research will be necessary to determine the practical role of these investigations in the management and progress of the complications of type 1 diabetes.

**Trial registration:**

Clinical Trial number NCT01279928, ClinicalTrials.gov

## Background

Type 1 diabetes is a known cause of microvascular damage, which leads to eventual capillary obliteration. Current techniques detect damage to the cardiovascular system once disease is well established. This pilot study evaluated the use of other more recent investigation techniques: nailfold capillaroscopy, laser Doppler flowmetry, retinal vessel analysis and 24-hour ambulatory blood pressure monitoring (24-hr ABPM) - in a paediatric and adolescent population with type 1 diabetes, in determining early microvascular changes.

**Figure 1:**
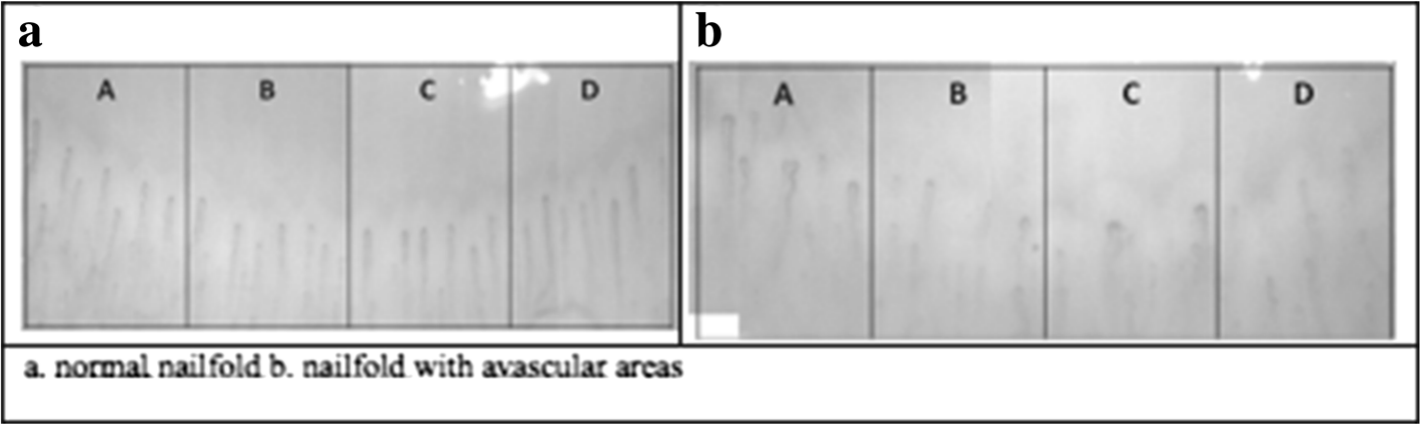
**Normal vs abnormal nailfold capillaroscopy.** Image **a**. demonstrates a normal image of a nailfold capillary bed with regular spacing between capillaries. Image **b**. shows irregular capillary spacing with gaps between the capillary loops.

Nailfold capillaroscopy visualises the capillary network and is a non-invasive painless technique. The capillary bed in the nailfold is both accessible and revealing: capillaries lie parallel to the skin allowing the whole capillary loop to be visualized (Figure 1). There are different capillary abnormalities which can occur at the nailfold, including:

• Normal patterns of capillary vessels that look like hairpins and are regularly placed along the nailfold [1-3].

• Loss of capillaries and/or avascular areas – a decreased number of loops (<30 of 5 mm in the distal row of the nailfold), or the loss of two contiguous capillaries [4,5].

• Giant capillaries – These are considered a major abnormality [6] and appear as homogenously enlarged loops with a diameter >50 mm and are symmetrical in shape [4,5].

• Microaneurysms – irregularly enlarged, circumscribed increase of capillary diameter [4,5].

• Angiogenesis – highly tortuous or branched capillary loop clusters, surrounded by dropout of normal capillary loops.

• Local microhaemorrhages – associated with early vascular damage. Shape is variable [4,7], but their presence is highly reproducible and repeatable and they are virtually absent in the healthy population [6].

• Modified architectural derangement – A combination of the above: capillary architectural derangements are an early feature of microangiopathy [4].

Several capillary changes have been described in diabetes with poor metabolic control [1,3,8]: however, not all studies have found these changes [9]. Some studies have examined changes in a paediatric population [2]; however, the specific morphological abnormalities in children have not been well-defined.

Laser Doppler flowmetry measures the cutaneous microvascular blood flow. As well as measuring baseline values, three provocations were used to produce vasodilatation and reperfusion: the iontophoresis of acetylcholine, post-occlusive hyperaemia, and thermal hyperaemia. In a paediatric and adolescent population, perfusion response differences between patients with type 1 diabetes and controls have been shown, including a decreased response to acetylcholine iontophoresis and thermal hyperaemia [10].

The development of semi-automated computer-based retinal imaging programs has stimulated research in the field of retinal vessel analysis, which has research potential in the diabetic field. Studies have shown an association between a wider arteriolar diameter and retinopathy in adolescents with type 1 diabetes [11]. This project investigated the retinal vessel diameter (both arterioles and venules) and the association with both clinical outcomes and the other techniques investigated in this study.

24-hour ambulatory blood pressure monitoring (24-hr ABPM) is an alternative to the traditional measurement of clinic blood pressure (BP) monitoring. It allows BP readings to be taken regularly throughout a 24-hour period. These readings form a 24-hr BP profile, which consists of the average day and night systolic and diastolic BP as well as the diurnal day-night ratio (or BP dip). 24-hr ABPM is widely used clinically in the management of type 1 diabetes. A decrease in BP dip has been previously associated with microvascular damage in patients with type 1 diabetes [12].

Our objective, therefore, was to use these techniques in a pilot study to characterize microvascular changes – in a paediatric cohort with type 1 diabetes of moderate duration.

## Methods

We conducted a cross-sectional study of children and adolescents with type 1 diabetes, aged 8–18 years old between February and October 2010. Participants were approached at their regular endocrinologist appointment at the John Hunter Children’s Hospital. All patients aged 18 years or under with type 1 diabetes were eligible for this study, unless they had other autoimmune conditions. Tenets of the Declaration of Helsinki were followed; the Hunter New England Human Research Ethics Committee granted institutional review board approval, and written informed consent was obtained from all participants and/or their parents as appropriate.

Participants attended a three-hour morning session in which nailfold capillaroscopy and laser Doppler flowmetry were performed and 24-hr ABPM was commenced; following this a second session was completed where the 24-hr ABPM was removed and retinal images were taken by a trained technician. Patients were asked to refrain from caffeine and nicotine for 12 hours prior to the investigations taking place. All investigations were carried out in a clinic room with a temperature controlled to 22-23°C. The STROBE statement (strengthening the reporting of observational studies in epidemiology) was followed, within the constraints of our study design.

### Nailfold capillaroscopy

The capillaroscopy technique using a capillaroscope camera (Basler Technologies), with Capiscope© software (KK Technology, Devon, England) has been described elsewhere [13]. Nailfold capillaroscopy was performed on the 2nd and 4th digit of both hands as a representation of capillary vasculature, with clinical restraints (including discomfort) limiting a fuller examination in this paediatric population [14,15]. The 4th finger of the non-dominant hand is thought to be the most representative of morphological features [16]. A panoramic video (100x magnification) was taken of the nailfold with the manual production of a single panoramic mosaic of each nailfold.

**Figure 2:**
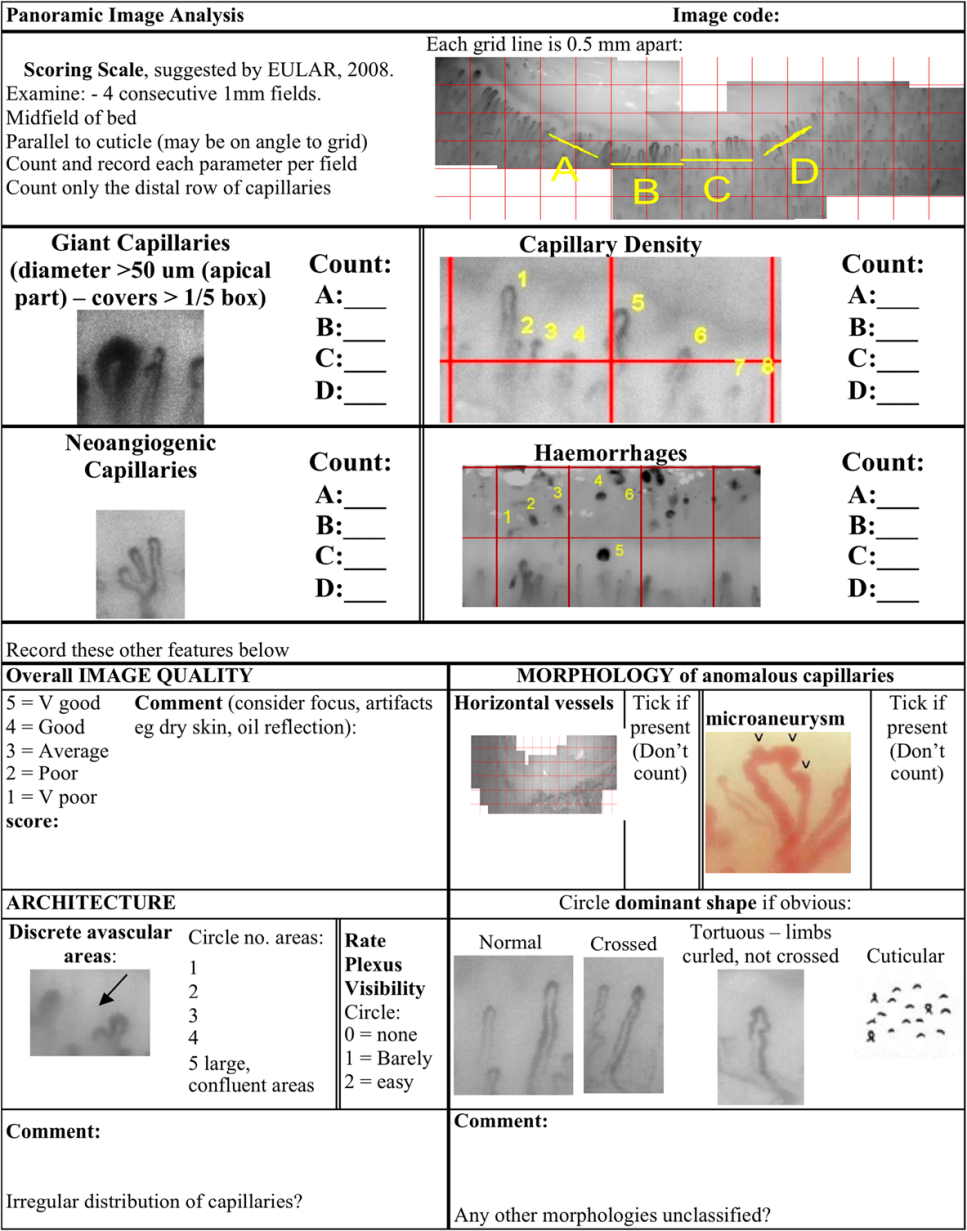
**Nailfold capillaroscopy image assessing tool.** Scoring scale developed by the authors, based on EULAR (European League Against Rheumatism) Guidelines (2008) [17]. Each nailfold image was divided into 4 consecutive 1 mm fields, which were analysed for nailfold abnormalities including: giant capillaries, capillary density, neoangiogenic capillaries, haemorrhages, morphology, presence of microaneurysms, avascular areas and the dominant capillary shape. The score was the average of each assessors abnormality count, for each nailfold.

The presence of morphological features - including giant capillaries, capillary density, neoangiogenic capillaries, micro-haemorrhages, micro-aneurysms and avascular regions - was assessed by the capillaroscope technician and two immunologists, who were blinded to the clinical history of each participant. The analysis determined the presence and number of each abnormality, and calculated the capillary density from each nailfold image (see Figure 2). An average score for each abnormality was calculated from the three assessors scores of four individual nailfolds.

### Laser Doppler flowmetry

The protocol used for laser Doppler flowmetry using a Periflux 5001 laser Doppler flowmeter (Perimed AB, Järfälla, Sweden) has been described elsewhere [18]. In this study skin perfusion was measured continuously on the volar aspect of the forearm. Peripheral microvascular blood flow results were digitally recorded, and analysed offline using custom software (Perisoft 2.1, Perimed AB, Järfälla, Sweden), by one investigator blinded to patient details [19]. Variables recorded included the baseline perfusion for both the iontophoresis probe and the temperature probe and the peak from the 6th dose of ACh. Finally, the post-occlusive reactive hyperaemia and the peak temperature perfusion reading were determined.

### Retinal vessel assessment

Each participant’s retinal photographs were taken by a qualified retinal photographic technician using a Torcon (TRC-NW100) retinal camera during the final clinic visit. Two images of each eye were obtained, one focused on both the optic disc and macula, and the other the macula.

Analysis of the retinal vessel pictures was done by RetVIC (Centre for Eye Research Australia, Melbourne, Victoria). All pictures were analysed using semi-automated computer programs with trained graders (blinded to the clinical history of the participants) using set protocols, as previously described [20,21]. Briefly one disc-centered digitized image was used for this measurement. For each photograph, all arterioles and venules coursing through an area one-half to one disc-diameter from the optic disc were measured and diameters of the largest six arterioles and six venules were averaged to become the arteriole diameter and the venule diameter [21] variables.

### 24-hr ambulatory blood pressure monitoring

Ambulatory blood pressure monitoring was performed using Meditech ambulatory blood pressure monitors (ABPM-04, ABPM-05, Budapest, Hungary). Measurements were taken every 30 minutes during daytime (6 am to 10 pm) and hourly overnight. Results were recorded and analysed using CardioVisions v 1.14 (Meditech, Budapest, Hungary). Outlying blood pressure measurements (> 200 systolic mmHg and < 50 diastolic mmHg) were excluded from analysis. Age- and gender-based normal criteria [22] were used to find abnormal daytime, nighttime and 24-hour blood pressure definitions for normal systolic and diastolic blood pressure, subjects greater than 16 years of age were measured against adult references [23]. The presence of pre-hypertension and hypertension was determined on clinic and ambulatory blood pressure levels in association with systolic blood pressure load (percentage of systolic blood pressure readings above the 95th centile for gender and age) using standard definitions [22]. The BP dip was measured as mean daytime systolic blood pressure – mean nighttime blood pressure/mean daytime blood pressure x 100, with normal considered to be greater than 10%. The presence of masked hypertension (normal clinic BP but abnormal 24-hr ABPM) [24] was also recorded. All results were analysed by a cardiologist blinded to the participant’s medical history.

### Standard clinical parameters

Standard clinic blood and urine tests were conducted, and the presence of microvascular damage shown by traditional investigations established. Average HbA1c (calculated from the values entered into the paediatric diabetes database since admission to the clinic) and most recent HbA1c, were analysed for each participant using a DCA Vantage™ Analzer, (Seimens). The presence of known microvascular complications was established through previous diagnosis by a paediatric endocrinologist or by standard investigations. The presence of retinopathy (microaneurysms, haemorrhages, microvascular abnormalities, constriction or tortuosity of vessels) was determined from fundal photographs reviewed by an ophthalmologist and paediatric endocrinologist. Renal microvascular changes were diagnosed by the presence of microalbuminuria (Based on three timed overnight urine collections - > 20 mcg/min or Albumin/Creat. Ratio >2.5 mg/mmol) either during the research investigations or since the diagnosis of type 1 diabetes. Duration of type 1 diabetes since diagnosis was obtained, both as a continuous variable and whether a significant duration of type 1 diabetes (>8 years) was associated with microvascular changes detected by the investigations outlined above.

### Statistical analyses

Statistical analyses were performed using Stata version 10 (Stata Corporation, College Station, Texas, USA) and JMP Version 5.1 (SAS, Cary, North Carolina, USA). Variables were assessed for normality and found to display a normal distribution. Participant characteristics were summarised as mean ± SD. Clinical and measured variables were summarized as means (with 95% CIs) for continuous data, and as proportions (with percentages) for categorical information. Differences between these summary statistics were analysed using the Student *t* for continuous variables. Categorical variables were analysed using [chi]^2^ test or Fisher exact test as appropriate.

Linear regression analysis was used to correlate measured parameters. Pearson product–moment correlation coefficients and significance levels were calculated. A probability value of p < 0.05 (two-sided), was considered significant.

## Results

26 participants were recruited for this study (13 males). The mean age of participants was 14.3 ± 2.3 years and the mean duration of type 1 diabetes of was 7.9 ± 3.4 years. Participants had a mean HbA1c of 8.1 ± 1.1%. All participants completed the protocol and there were no adverse effects. Microvascular changes found on standard investigations were observed in four participants, one with signs of retinopathy and three with microalbuminuria. Participant baseline characteristics are shown in Table 1.

**Table 1:** Patient baseline characteristics

**N**	**26**
Age	*14.3 ± 2.26
Gender (% male)	50
BMI (kg/m^2^)	23.7 ± 5.0
Diabetes duration (years)	7.9 ± 3.4
HbA1c (%) – most recent	8.9 ± 1.7
HbA1c (%) - average	8.1 ± 1.1
Insulin pump (% which use)	50
Total/HDL ratio	3.1 ± 0.7
Albumin/Creatinine ratio (mg/mmol)	2.0 ± 4.0
Clinic Systolic BP (mmHg)	109.6 ± 9.3
Clinic Diastolic BP (mmHg)	72.0 ± 7.9
Participants with signs of retinopathy (n)	1
Participants with microalbuminuria (n)	3

*All values presented as mean ± SD.

### Nailfold capillaroscopy

Key nailfold capillaroscopy parameters were stratified by clinical outcomes (Table 2). Avascular areas were significantly more common in participants with type 1 diabetes complications (t = -2.33, p = 0.03). Recent HbA1c was positively associated with the number of microhaemorrhages (r = 0.44, p = 0.03).

**Table 2:** Nailfold capillaroscopy, laser Doppler flowmetry, retinal vessel and 24-hr ABPM parameters and effect of gender, microvascular complications and average HbA1c

	**Gender**			**Microvascular Complications**			**Average HbA1c (%)**		
**z**	**Male**	**Female**	**p=**	**Absent**	**Present**	**p=**	**≤8**	**>8**	**p=**
n	13	13		22	4		13	13	
NC*: Avascular areas (number/nailbed)	1.0 (0.5-1.4)	1.1 (0.9-1.2)	0.74	0.9 (0.7-1.1)	1.6 (0.8-2.3)	**0.03**	0.9 (0.5-1.2)	1.2 (0.8-1.5)	0.22
NC: micro-aneurysms (number/nailbed)	0.5 (0.3-0.6)	0.6 (0.5-0.7)	0.06	0.5 (0.4-0.6)	0.7 (0.0-0.0)	0.20	0.5 (0.4-0.6)	0.6 (0.4-0.7)	0.55
Laser Doppler flowmetry: Baseline perfusion (PU^1^)	19.1(10.3-3.4)**	19.1 (11.6-26.6)	1.00	20.6 (14.2-27.1)	10.5 (6.8-14.3)	0.19	18.3 (9.3-27.3)	19.9 (12.7-27.1)	0.78
Retinal vessel analysis: Arteriole-to-venule ratio	0.7 (0.7-0.7)	0.7 (0.7-0.7)	0.56	0.7 (0.7-0.7)	0.07 (0.7-0.8)	0.41	0.7 (0.7-0.7)	0.7 (0.7-0.7)	0.96
24-hr ABPM: BP Dip	7.1 (3.56-10.6)	9.2 (6.6-11.8)	0.32	7.8 (5.4-10.2)	10.1 (4.0-16.1)	0.46	7.4 (3.6-11.2)	8.9 (6.7-11.2)	0.48

*NC (Nailfold Capillaroscopy) **Values presented as mean (95% CI) ^1^Perfusion units.

### Laser Doppler flowmetry

A number of different variables were associated with the individual laser Doppler flowmetry results. Age (r = 0.40, p = 0.04) and diabetes duration (r = 0.4, p = 0.03) had a significant inverse association with baseline perfusion, (Table 2). However, no significant difference was shown between participants with type 1 diabetes duration less than 8 years and those with diabetes duration greater than 8 years (t = 0.9, p = 0.4). When laser Doppler flowmetry results were compared with nailfold capillaroscopy results it was found that participants with decreased baseline perfusion had increased capillary density (r = 0.63, p = 0.001) and were more likely to have microaneurysms (r = 0.40, p = 0.04). Other variables associated with laser Doppler flowmetry and nailfold capillaroscopy were not significantly associated with standard clinical parameters or microvascular investigations.

### Retinal vessel analysis

Retinal venule diameter was significantly greater in participants with hypertension (r = 0.45, p = 0.02). Arteriole diameter (t = 2.6, p = 0.02) was significantly increased in participants with a long duration of diabetes (>8 years). Arteriole-to-venule ratio was stratified by clinical outcomes (Table 2). No other statistically significant associations were present.

### 24-hr ambulatory blood pressure monitoring

54% of this cohort had a BP dip of less than 10%. Table 2 shows BP dip stratified by clinical outcomes. The mean of the 24-hr systolic BP was 118.4 mm Hg (CI 95% 114.7-122.0) and the 24 hr diastolic BP was 66 mmHg (CI 95% 63.7-68.3). Five participants had either pre- or masked-hypertension on 24-hr ABPM.

## Discussion

This pilot study has demonstrated that results from nailfold capillaroscopy, laser Doppler flowmetry and retinal vessel analysis were significantly linked to type 1 diabetes-related microvascular damage, such as retinopathy and the presence of microalbuminuria, as well as longer diabetes duration and higher HbA1c results.

Morphological changes were seen on nailfold capillaroscopy. More avascular areas were found in participants with evidence of microvascular disease (presence of retinopathy or microalbuminuria), and participants with a recently elevated HbA1c had an increased number of microhaemorrhages. These findings are consistent with previous studies in adults [1,25], and their detection in our paediatric population is a novel finding. This result suggests that changes to the microcirculation in the periphery are linked to type 1 diabetes end-organ microcirculatory damage.

Participants with a longer duration of diabetes had decreased baseline perfusion of the forearm skin. This differs from reports in adults, which found either increased baseline perfusion associated with HbA1c >7.5% [26], or no difference in baseline perfusion between participants with type 1 diabetes and controls [10]. Vascular heterogeneity could have caused this effect as these studies took the perfusion measurements on the dorsum of the foot [27]. The changes in adults are at least partly related to significant autonomic disturbances [28], which become more common as duration of diabetes increases [29]. Autonomic disturbances develop in children with diabetes and become more pronounced with aging [30]. Our findings, indicating microvascular changes antedating development of autonomic neuropathy, are consistent with earlier pathological involvement of vessels than nerves, and/or an ascending neuropathic process [31]. The association between decreased perfusion with diabetes duration could be due to functional changes as a consequence of long duration of diabetes. However, age was also associated with decreased baseline perfusion, possibly confounding this result. A larger study is indicated to pursue these important relationships further.

Both morphological and functional capillary changes were found in participants. Participants’ with decreased baseline perfusion had increased capillary density, and an increase in the number of microaneurysms. This suggests that while there may be an increase in the number of capillaries, they are dysmorphic and therefore have less efficient blood flow (as shown by decreased perfusion). These associations are not well documented in the literature, with a single article describing an association between nailfold morphological changes and functional flowmetry changes [32] Alternatively, as poor flow may be implicated in the hypoxic origin of the local nailbed changes, the decreased basal flow could be directly implicated in the ontogeny of the capillaroscopy changes.

Our study confirmed literature reports that hypertension is associated with increased retinal venule diameter [33]. We found that larger arteriolar diameter i associated with longer diabetes duration; other studies have found arteriolar diameter to be associated with a risk retinopathy development [11] although not diabetes duration. It is thought that arteriolar dilation is a direct indicator of retinal microvascular dysfunction in diabetes; the physiological basis of this has been widely explored in other studies [11,34]. Hypertension is more common in children with type 1 diabetes, with 13.9% of adolescents with a BP >97th centile [35].

A review of the literature found two studies, which had similar findings to our research. The first, in adults, found nailfold capillary abnormalities were present in a higher frequency in participants with type 1 diabetes compared to those with type 2 diabetes or no diabetes and these abnormalities correlated with presence of retinal damage [36]. Another adult study by Nguyen et al. found the presence of diabetic retinopathy was associated with a reduction in skin microcirculation response to iontophoresis of ACh [37]. These results are supportive of our findings, although both were studies in adults and did not include all the micro- and macro-vascular investigations we performed. Our detection of similar findings in children implies that changes develop early in the disease, potentially highlighting the importance of early diagnosis and intervention.

This study suggests that these investigations are associated with the traditional markers of poor diabetic control and consequently microvascular complications. These results also suggest that there are common microvascular changes throughout the body with results of different investigations in concordance – specifically those examining capillaries in the nailfold and functional skin perfusion changes. The finding that microvascular change is widespread is supported by past research [3,33]. These changes were also found in a paediatric population with a relatively short duration since diagnosis, suggesting that microvascular changes start early in the disease and are present before clinically detectable microvascular disease.

The strength of this study was its concurrent novel use of four different investigations in the same population, with all participants completing the study. The major limitation of this project was that no control group was studied. However, there have previously been studies in the literature, looking at these techniques in normal children in a similar age group and ethnicity to our research participants. A study of 329 school-aged children with nailfold capillaroscopy by Terreri et al. [38] found that avascular areas were rare in normal children (found in 2% of their cohort) and that capillary density increased with age: the methods of calculations used were comparable to ours. Although we did not find a similar correlation between capillary density and age, the lack of avascular areas found in a normal cohort and those in our population with well-controlled type 1 diabetes, suggests this should be pursued in larger studies in the future.

Other limitations include this study’s cross-sectional nature; temporal relationships between study variables were unable to be determined. Subsequent studies should be of a prospective longitudinal design to determine whether children with changes in these parameters progress to develop clinically significant microvascular disease. Our data suggests research in this field should focus on type 1 diabetes and nailfold capillaroscopy, baseline perfusion and ACh perfusion response (from laser Doppler flowmetry). In addition, nailfold capillaroscopy is non-invasive and gives direct visual feedback to young patients with type 1 diabetes. The immediacy of the process can potentially have a more powerful impact when explaining diabetes complications [39] and thus potentially influence diabetic control, which in turn influences the outcomes [40].

## Conclusions

Nailfold capillaroscopy, laser Doppler flowmetry and retinal vessel analysis detected microvascular changes in participants with poorly controlled type 1 diabetes (determined by HbA1c, presence of retinopathy and microalbuminuria). This raises the possibility that type 1 diabetes associated microvascular complications may be detected earlier by methods other than those currently employed. It is hoped that the results from this exploratory study will be used as a basis for the development of methods that will allow trials in the earlier detection and monitoring of microvascular changes in type 1 diabetes. Ultimately, this may delay the progression of this debilitating disease.

## Abbreviations

24-hr ABPM: 24-hour ambulatory blood pressure monitoring; BP: Blood pressure; ACh: Acetylcholine.

## Competing interests

The authors declare that they have no competing interests.

## Authors’ contributions

S.H. collected and organised data on nailfold capillaroscopy, laser Doppler flowmetry, retinal vessel analysis and 24-hr ABPM and wrote the manuscript. R.B. analysed nailfold capillaroscopy data and reviewed/edited the manuscript. P.A.C. analysed the retinal images, reviewed/edited the manuscript and contributed to the discussion. I.W. provided laser Doppler training, contributed to the discussion and reviewed/edited the manuscript. M.S. collected data on nailfold capillaroscopy and reviewed manuscript. G.R. analysed nailfold capillaroscopy data, performed statistical analysis and reviewed/edited the manuscript. All authors read and approved the final manuscript.

## Pre-publication history

The pre-publication history for this paper can be accessed here:

http://www.biomedcentral.com/1472-6823/13/41/prepub
